# Genes and elements involved in the regulation of the nervous system and growth affect the development of spinal deformity in *Cyprinus carpio*

**DOI:** 10.1371/journal.pone.0266447

**Published:** 2022-04-08

**Authors:** Zoltán Bagi, Katalin Balog, Bianka Tóth, Milán Fehér, Péter Bársony, Edina Baranyai, Sándor Harangi, Mohammad Reza Ashrafzadeh, Bettina Hegedűs, László Stündl, Szilvia Kusza

**Affiliations:** 1 Centre for Agricultural Genomics and Biotechnology, Faculty of Agricultural and Food Sciences and Environmental Management, University of Debrecen, Debrecen, Hungary; 2 Doctoral School of Animal Science, University of Debrecen, Debrecen, Hungary; 3 Department of Animal Husbandry, Laboratory of Aquaculture, Institute of Animal Science, Biotechnology and Nature Conservation, Faculty of Agricultural and Food Sciences and Environmental Management, University of Debrecen, Debrecen, Hungary; 4 Department of Animal Nutrition and Food Biotechnology Faculty of Agricultural and Food Sciences and Environmental Sciences, University of Debrecen, Debrecen, Hungary; 5 Department of Inorganic and Analytical Chemistry, Atomic Spectroscopy Laboratory, University of Debrecen, Debrecen, Hungary; 6 Department of Fisheries and Environmental Sciences, Faculty of Natural Resources and Earth Sciences, Shahrekord University, Shahrekord, Iran; 7 Institute of Genetics and Biotechnology, Hungarian University of Agriculture and Life Sciences, Gödöllő, Hungary; 8 Institute of Food Technology, Faculty of Agricultural and Food Sciences and Environmental Management, University of Debrecen, Debrecen, Hungary; Karlsruhe Institute of Technology, GERMANY

## Abstract

Spinal deformity is a serious economic and animal welfare problem in intensive fish farming systems, which will be a significant unsolved problem for the fish sector. The aim of this study was to determine the relative expression of genes (Akt1 substrate 1, Calreticulin, Collagen type I alpha 2 chain, Corticotropin-releasing hormone, Chromodomain-Helicase DNA-binding, Growth hormone, Insulin like growth factor 1, Myostatin, Sine oculis-related homeobox 3, Toll-like receptor 2) in different tissues associated with spinal deformity and to determine the macroelement (calcium, magnesium, phosphorus, potassium, sodium, sulfur) and microelement (barium, copper, iron, manganese, strontium, zinc) content of spine in healthy and deformed common carps (*Cyprinus carpio*) in Hungary. The mRNA levels of the genes were measured in 7 different tissues (abdominal fat, blood, brain, dorsal muscle, genitals, heart, liver) by qRT-PCR. Correlations between gene expression and element content were analyzed by using linear regression and Spearman rank correlation. In a total of 15 cases, we found a statistically significant connection between gene expression in a tissue and the macro- or microelement content of the spine. In these contexts, the genes Akt1 substrate 1 (3), Collagen type I alpha 2 chain (2), Corticotropin-releasing hormone (4), Insulin-like growth factor 1 (4), and Myostatin (2), the tissue’s blood (3), brain (6), heart (5), and liver (1), the macroelements sodium (4), magnesium (4), phosphorus (1) and sulfur (2) as well as the microelement iron (4) were involved. We also found statistically significant mRNA level differences between healthy and deformed common carps in tissues that were not directly affected by the deformation. Based on our results, genes regulating the nervous system and growth, elements, and tissues are the most associated components in the phenomenon of spinal deformity. With our study, we wish to give direction to and momentum for the exploration of these complex processes.

## Introduction

The common carp (*Cyprinus carpio*) is natively distributed from the Danube River to the Black, Caspian and Aral Sea basins. In Europe, common carp has apparently been domesticated since the Middle Ages and wild populations of the Danube are assumed to be the origin of the cultivated stocks [1]. The species is a widespread and economically important species in natural waters and hatcheries, and has enormous commercial importance and market demand.

Fish species may exhibit various kinds of morphological deformities, such as bone and skin neoplasms and spine deformations [2], dysplasia in the opercular bones, mandibular bones and maxillary-mandibular apparatus and anomalies in fins and eyes [3]. Skeletal deformities are a major factor that affects the external morphology, survival and growth of the fish and production cost and so downgrade hatcheries’ production [4]. Spinal deformity has been observed in a large number of vertebrates and has been reviewed in several studies. Spinal lesions have also been observed in wildlife such as sand tiger shark (*Carcharias taurus*), small-mouthed perch (*Micropterus dolomieui*) and brown bear (*Ursus arctos*), often reflecting environmental problems [5–7]. Furthermore, it is often described in intensive production systems, because in domesticated animals such as broilers, pigs and farmed fish it is a recurring problem that poses a number of economic challenges [8, 9], in addition to emerging animal welfare concerns [10–13]. Thus, the success and development of a sustainable aquaculture sector also depend on how these common diseases are treated, as fish diseases also have a serious impact on the economic situation of farmers, production losses, investment losses and loss of consumer confidence [14]. In 2006, the World Bank reported an annual loss of $ 3 billion in aquaculture due to disease, prevention and health treatments [14]. Real economic losses are difficult to estimate, but the estimated minimum annual loss in European aquaculture is more than € 50 million per year and it is assumed that 50% of this is due to skeletal deformities and abnormalities [15].

Many factors may play an important role in the appearance of the spinal deformity disorder, such as non-hereditary congenital disorders [16], breeding and feeding techniques [17, 18], traumatic injuries [19], environmental disturbances and toxic substances (including heavy metals) [16, 20–22], furthermore parasites [23, 24] and some pathogenic bacteria [25] as well as hereditary genetic causes [26]. For these reasons, there is considerable interest in exploring the possible causes and treatments of deformities [27].

Several studies [28–32] examined the effect of different genes on the development of spinal deformity, whereas the genetic background of the disease remains unclear. Many genes directly or indirectly affect the formation and function of the skeletal system, which is why several studies have been performed on the mechanisms of bone development in mammals and bony fish [29, 33, 34]. Molecular and cellular mechanisms involved in different developmental pathways, and genetic factors regulating stem cell identification and differentiation have been found to be conserved at the molecular level among vertebrates in terms of both sequence and expression pattern [20, 35]. The most common spinal malformations in fish are mainly dorso-ventral anomalies (kyphosis and lordosis) or coronal plane curvature (scoliosis), which may be associated with variability [36], thus becoming unacceptable to consumers [13, 37].

Examining the key factors in bone development (genes, macroelements and microelements) and their possible relationships may provide a new approach to research into the phenomenon of spinal deformity. In our work, relative expression level of the following spinal deformity related genes: Akt1 substrate 1 (Akt1s1), Calreticulin (CALR), Collagen type I alpha 2 chain (COL1A2), Corticotropin-releasing hormone (CRH), Chromodomain-Helicase DNA-binding (CHD), Growth hormone (GH), Insulin like growth factor 1 (IGF1), Myostatin (MSTN), Sine oculis-related homeobox 3 (SIX3), Toll-like receptor 2 (TLR2) were determined in blood, heart, liver, genitals, dorsal muscle, abdominal fat and brain tissues of normal and spinal deformed common carp individuals raised from the same hatchery and kept using the same technology.

Akt1s1 gene is the center of a complex signaling pathway that is one of the most intensively studied pathways today [38], because it plays a key role in cellular metabolism, growth, proliferation, motility, survival, and apoptosis [39, 40]. Calreticulin is a highly conserved protein that is primarily a nurse protein of the endoplasmic reticulum (ER) that is involved in many cellular processes, such as repairing misfolded proteins (conformational folding), but also plays a role in cell adhesion [28]. CALR is an autoantigen that has a strong signal value due to any change in the body, such as inflammation or other stressors [41, 42].

Collagen, the most abundant extracellular matrix protein in the animal kingdom belongs to a family of fibrous proteins, which transfer load in tissues and provide a highly biocompatible environment for cells [43]. Mutations in the COL1A2 gene lead to a variety of connective tissue disorders [44]. Type I collagen is a major structural protein in both bone and skin; mutations in type I collagen genes cause bone disease. Mutant collagen may be expressed differently in bone and skin [44]. Type I collagen (transcribed from the COL1A1 and COL1A2 genes) is important for maintaining vascular wall elasticity and is a critical part of the extracellular matrix (ECM) [43].

The corticotropin-releasing hormone (CRH) is synthesized in the paraventricular nucleus (PVN) of the hypothalamus, from where the pituitary portal enters the circulation to coordinate pituitary-adrenal stress responses. Thus, CRH gene is primarily a regulatory gene responsible for stress responses expressed in the brain. In 2008, Kulkarni et al. [45] examined the chromodomain helicase DNA-binding (CHD) protein family and found that CHD7 may be responsible for the development of idiopathic scoliosis [45]. Mutations in the protein family can cause a number of deformities and diseases.

Growth is a polygenic and environmentally regulated trait, whose most influential genes are those encoding the growth hormone (GH) and the insulin-like growth factor-I (IGF1), since these are the center of the hypothalamic-pituitary-somatotropic (HPS) axis [46]. The HPS axis is one of the most important endocrine systems that functionally and structurally regulates skeletal and soft tissue development as well as growth-related processes. In general, IGF1 is predominantly synthesized in the liver and is an important mediator of growth hormone effects during postembryonic life [47]. Stimulation of IGF1 expression by GH in the brain indicates that IGF1 may mediate GH central nerve function in common carp [46]. MSTN (myostatin) is a member of the transforming growth factor-β superfamily (TGF-β superfamily) that negatively adjust skeletal muscle development and growth, by inhibiting cell cycle progression [48].

The SIX homeobox 3 (SIX3) gene is responsible for the normal development of the pituitary gland in mammals and its genetic variation or absence causes hypopituitarism, suggesting that this gene is a potential candidate for association with growth characteristics in animals [49]. TLR2 recognizes various components of many microorganisms. Gram-negative bacteria (such as *Mycoplasma* and *Spirochaeta lipoproteins*), the peptidoglycan of Gram-positive bacteria, and TLR2 also play an important role in the recognition of endogenous ligands produced during inflammation, such as HSP60 and HSP70 [50, 51].

Further analyses were performed on macroelement and microelement contents in the spinal column of the same individuals. Macro- and microelements are essential for the proper functioning of the physiological processes of any organism [52]. The macroelements such as calcium (Ca), magnesium (Mg), phosphorus (P), potassium (K), sodium (Na) and sulfur (S). The microelements, also known as trace elements, such as barium (Ba), copper (Cu), iron (Fe), manganese (Mn) and strontium (Sr), which are present in the body in a percentage smaller than are macroelements, but are equally essential [53, 54]. On the other hand, excessive amounts of trace elements can also have harmful effects on the body [52, 55, 56]. The most common heavy metals detectable in fishes are cadmium (Cd), lead (Pb), mercury (Hg), zinc (Zn), copper (Cu), nickel (Ni), cobalt (Co), molybdenum (Mo), chromium (Cr) and tin (Sn) [22]. Of these, Cd, Pb, Hg, Zn, and Cr were the most studied for the fish deformity [52]. The most bioavailable form of metals that can cause poisoning is the dissolved ionic form. Significant toxicity of metals in fishes can result from organic forms of the metal, which can affect many physiological systems. The extent of toxicity depends only on its bioavailability and its toxicokinetics (absorption, distribution, biotransformation and excretion) and toxicodynamics (interactions with ligands) [56]. In fish, minerals also play an important role in osmoregulation and skeletal formation, as well as in intermediate metabolic processes. It is difficult to study mineral concentrations adequate or harmful for fish, because some minerals are needed in very small amounts, whereas others are absorbed in significant amounts from water through the gills as well as from feed [22, 56]. Feeding defects can lead to severe mineral deficiencies, which can lead to decreased resilience, anemia, or even skeletal deformity in fish [57, 58]. The absence of microelements can also be dangerous, as traces of Zn, Cu, Fe, and Se are required for metalloenzymes, which are key to maintaining cellular functions in the immune system [59]. The functions and effects of the macro- and microelements included in the study are summarized in Table 1.

**Table 1:** Summary of the functions and deficiency symptoms/-diseases of the macro- and microelements used in the study.

Macro- and microelements	Function	Deficiency symptoms/-Diseases
**Ca**	Role in specific signaling and regulatory mechanisms of neurons [60]. Ca increase promotes bone formation and inhibits bone resorption [61].	Decrease in calcium levels can cause tetany, and the trunk in the brain can become calcified.
**Mg**	Enzyme cofactor, plays an important role in the structure of the solid skeletal system, osmoregulation, and neuromuscular transmission [62].	Lordosis, poor growth and protein production, as well as degeneration of muscle tissue [63].
**P**	It is found in all cells, plays a vital role in metabolic processes, and is a component of bones, teeth, adenosine triphosphate (ATP), and nucleic acids [62, 64].	Deterioration of the condition of the bones, as well as muscle and joint problems and pain can occur.
**K**	Regulates water and acid-base balance in the blood and tissues. Active transport of potassium is key in cardiovascular and nervous system function [65].	Lack of it can lead to muscle weakness, breathing problems and arrhythmias.
**Na**	Sodium can affect fluid balance and muscle function, it is important in nerve transmission processes in the nervous system [66].	Its deficiency causes an imbalance and increased water uptake by the tissues, leading to swelling.
**S**	It regulates acid-base balance, preserves cell permeability, and activates nerve and muscle function [64, 67, 68].	Slow wound healing, scarring and arthritis may develop.
**Ba**	Barium compounds are easily absorbed in the lungs or gastrointestinal tract, but after absorption, barium accumulates in the bones and barium permanently stimulates muscles [69].	It can cause vasoconstriction and acute poisoning can also cause arrhythmias or skeletal muscle paralysis.
**Cu**	It is essential for the proper functioning of the respiratory chain, the synthesis of hormones, neurotransmitters, and the stabilization of the extracellular matrix [70].	Fat metabolism disorders, bone marrow failure and degenerative nervous system diseases may also occur.
**Fe**	Iron is involved in oxygen transport [62, 64] in electron transport, the synthesis of neurotransmitters, their uptake and degradation, which may directly or indirectly alter brain function and amino acid metabolism through reduction [71].	The most common disease is microcyte hypochromic anemia. It usually damages the nervous system, memory and thinking functions.
**Mn**	Manganese binds to mitochondria and is deposited in the liver. Excessive manganese exposure can cause congestion in the hepatic vein, creating focal necrotic areas.	Slower weight gain as well as increased liver and spleen mass may develop [72].
**Sr**	Strontium is chemically very similar to calcium, deposited in the bones, and stimulates bone building while reducing its breakdown.	The high doses of strontium can cause changes in mineralization, and can stimulate the bone formation [73].

The spinal column is an ideal target for examining spinal deformity for two reasons. On the one hand, this osseous tissue is directly affected in the deleterious lesion. On the other hand, one of the most important functions of this tissue type is to deposit macro- and microelements. The ion concentration in bone can reflect the chronic disorders of its metabolism and its distribution in the body. Ion imbalance can affect bone cells and change the structure and function of the skeleton [74]. Thus, elemental contents measured in the spinal column may indicate disorders and pathological changes in both bone tissues and other parts of the body.

The research hypotheses were that (1) there are differences between the gene expression levels we planned to study in normal and deformed groups; and (2) there is a correlation between the relative gene expression level of the genes studied and the micro- and macroelement content of their spinal column. When selecting the genes and the micro- and macroelements for the study, we considered how they exert their physiological effects in the tissues. Our results contribute to the study of common carp spinal deformity disease and may provide gap-filling data to explore the development of the disease in order to maintain competitive production.

## Materials and methods

### Experimental setup and rearing conditions

Twelve common carps were purchased from artificial propagation. Prior to the experiment, samples were reared under controlled conditions, with identical feeding and environmental parameters. Thus, external factors that could affect the individual growth differences such as applied diet, feeding rate, stocking density, amount and length of illumination [75], water temperature, pH [76], and other environmental factors were excluded.

Fish were kept in a recirculation aquaculture system (RAS), provided with mechanical and aerated biofilter and UV lamp. The water volume of the plastic tanks was 350 litres. All individuals were fed three times daily (08:00, 12:00 and 16:00) with commercially available dry feeds; the feeding rate was 2.5–4.0% of the total biomass [77]. Uneaten feed and faeces were removed daily. Oxygen saturation was maintained above 80% by aeration stones and the temperature was controlled at 25.05 ± 0.5 °C. The photoperiod was 12 h light and 12 h dark. Water temperature and dissolved oxygen were checked daily with a HACH HQ30d portable meter (HACH CO., Loveland, CO, USA). NO_2_^-^, NO_3_^-^ and NH_4_^+^ concentrations were measured using the HACH Lange DR/3900 spectrophotometer (HACH CO., Loveland, CO, USA). After 260 days post-hatch (dph), a total of 12 common carp individuals with an average weight of 253.95±18.97 g were randomly selected from the population and based on their phenotype and spinal deformity classified into following four groups: scaled common carps without spinal deformity (normal), mirror individuals without spinal deformity (normal), scaled common carps with spinal deformity (deformed), mirror individuals with spinal deformity (deformed).

Prior to the further analyses, fish were euthanized with clove oil solution [78]. Sample collection was approved and carried out in accordance with the local ethics committee’s guidelines of the University of Debrecen under the registration number: (DEMAB/15/2019).

### Quantitative analysis of relative gene expression levels

Total RNA was isolated from different tissues of common carp. Sampling was performed on day 260 of fish life. RNeasy Protect Animal Blood Kit (QIAGEN, Hilden, Germany) for blood, E.Z.N.A. Tissue RNA Kit (Omega Bio-tek, Norcross, USA) for heart, liver, and dorsal muscle, and E.Z.N.A.^®^ Total RNA Kit II (Omega Bio-tek, Norcross, USA) for brain, abdominal fat and genitals were used by following the manufacturer’s protocol. RNA quality and quantity were measured using a NanoDrop ND-1000 Spectrophotometer (Thermo Fisher Scientific, Waltham, MA, USA). Total RNA (100 ng) was reverse transcribed into cDNA with specific primers (Table 2) using the qPCR BIO cDNA Synthesis Kit (PCR Biosystems, London, United Kingdom). Quantitative real-time PCR (qRT-PCR) was performed using a 7300 Real-Time PCR System (Applied Biosystems, Foster City, CA, USA). The 10 μl reactions consisted of 5 μl PowerUp^*™*^ SYBR^®^ Green Master Mix (Applied Biosystems, Foster City, CA, USA), 0.6 μl each of 10 μM forward and reverse primers, 1.3 μl dH2O (Millipore Sigma, Burlington, MA, USA) and 2.5 μl cDNA (2 ng/μl). For each sample, quantitative PCR was performed in triplicate. Thermal cycling was performed under the following conditions: 95 °C for 10 min, 40 cycles of 95 °C for 15 s and 60 °C for 1 min. Forward and reverse primers for common carp (Table 2) were designed by Primer Express v3.0.1 software (Applied Biosystems, Foster City, CA, USA) and checked for target identity using National Center for Biotechnology Information (NCBI) Primer Blast [79].

**Table 2:** Data of applied primers.

Gene	Forward primer sequences (5’→3’)	Reverse primer sequences (5’→3’)	Amplicon length (bp)
β-actin2	CCATCGGCAATGAGCGTTTC	GCACAGCATAAGACTCACCCA	75
Akt1s1	AAGACCTCGCCTTAACACGG	CGCGCAAACATACATACGCA	78
CALR	AGGCAGAACCACCTAATCAA	CCACCTTCTCGTTGTCGATTT	121
COL1A2	ACATTGGTGGCGCAGATCA	ACTCCGATAGAGCCCAGCTT	191
CRH	ACCATAGTGCTGCTTTTCCATCA	AGTGATGACAGTTTTGCGCTTC	119
CHD	GAACAGGTCAGGGGTCGTT	GTAGGGTCTGTAGGTTGAGGC	82
GH	TCTTCTCATTTGGGAGTGTCTCA	AGACACCACTGTGAAATGTTCC	86
IGF1	CGCCTCGAGATGTATTGTGCAC	CTGTATGCCGTTGCGCTCGT	73
MSTN	ATCTACTTGTCCGGTGCGTG	ATGCATGTTCCAAAGCGTGC	91
SIX3	TTTAAAGAGCGGACGCGGAG	ATTTCCGACCTGTGTTGGTG	120
TLR2	TGTGTGCGACACTCCATTCA	GCACTAAGACAGCAGGGATCA	95

### Elemental content analysis of the spine

The whole spinal columns were dried in an oven to constant weight and digested with 2 x 4.0 ml of HNO_*3*_ (p.a. 65%). Then, 1.0 ml of H_2_O_2_ (p.a. 30%) was added to the samples, which were made up to 10 ml with Milli-Q water before the measurement. In total, 11 elements including six macroelements (Ca, Mg, P, K, Na, and S) and five trace elements (Ba, Cu, Fe, Mn, and Sr) were measured by using a 5110 VDV Agilent ICP-OES spectrometer (Agilent, Santa Clara, USA). A standard sample introduction system (Seaspray nebulizer and double pass spray chamber) was used for the measurement. Five-point calibration was used from a multi-element stock solution (Merck IV) and samples were introduced in triplicates. Data sets for macro- and microelements are available in (S1 Table).

### Statistical analysis

Relative gene expression values were normalized to β-actin and analyzed with the Pfaffl method [80]. Primer efficiency was calculated with LinRegPCR version 2017.0 software [81]. The average Ct values for each gene per tissue are shown in S2 Table. Statistical analyses were performed with SPSS 26.0 software (IBM Corp., Armonk, NY, USA). The normality assumption and homoscedasticity were assessed using the Shapiro Wilks and Levene tests, respectively. The independent samples T-test and Mann-Whitney U test were used to compare differences between two groups. Independent samples T-test was used when the test of homogeneity of variances (Levene statistic) was not significant, and if significant, Welch’s T-test was used. For non-normally distributed data, a Mann-Whitney U body was used.

In this study, a linear regression model was used to analyze the relationship between gene expression and element content in different tissue types. In cases where the mRNA expression or element content was not normally distributed, Spearman’s rank correlation test was applied to the analysis of the relationship. Spearman’s rank correlation is an alternative nonparametric method and performs similarly to linear regression [82].

## Results

### Quantitative analysis of relative gene expression levels in different tissues

**Fig 1:**
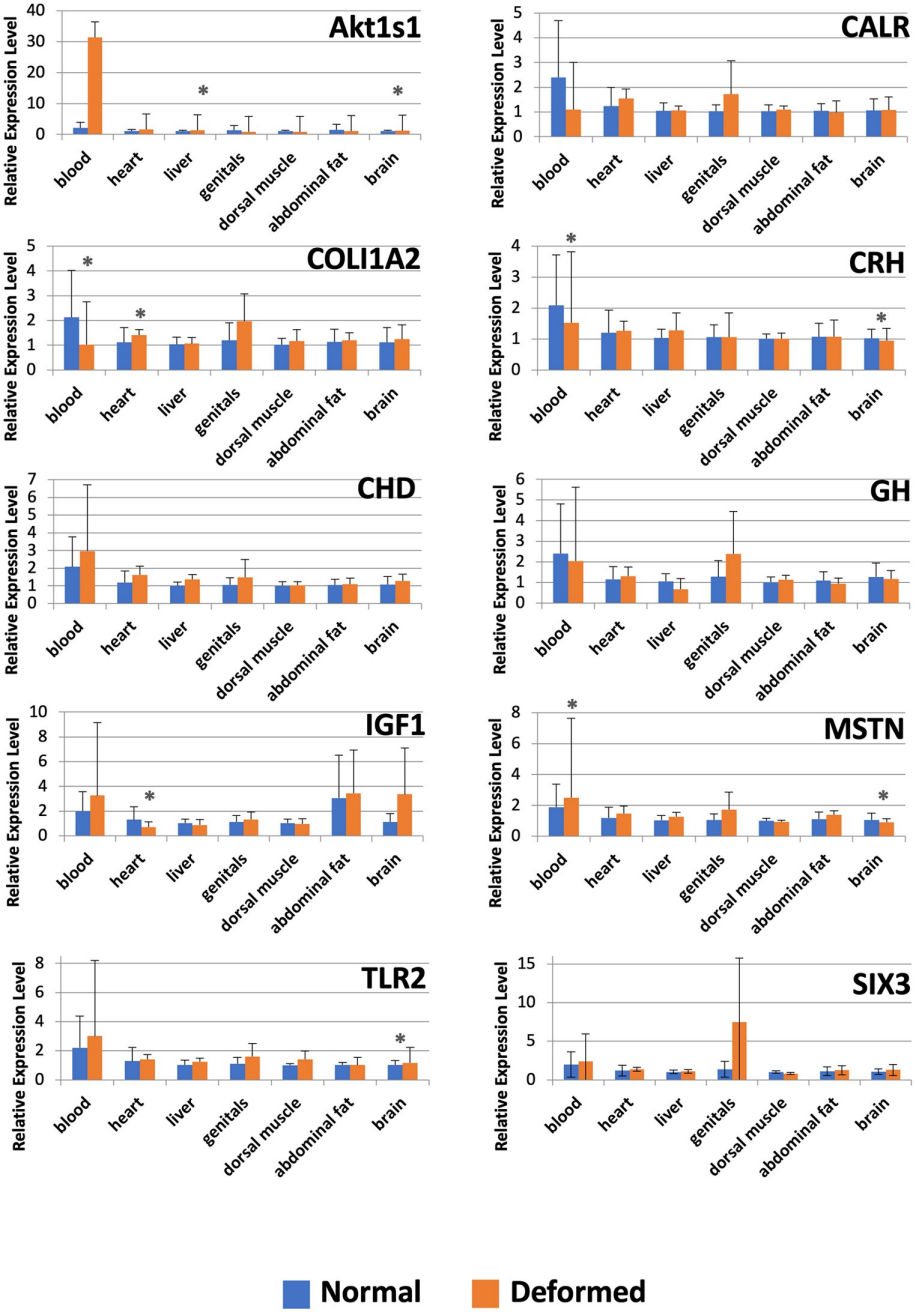
Relative gene expression levels in different tissues. * *p* ≤ 0.05.

The relative expression levels of Akt1s1, CALR, COL1A2, CHD, GH, IGF1, MSTN and SIX3 mRNA in seven tissue types are shown in Fig 1. These growth-related genes were usually expressed to different degrees in groups of normal and deformed fish.

The largest difference of mRNA levels was observed at Akt1s1 in blood, CALR in blood, IGF1 in brain and SIX3 in genitals. In general, the deformed group was characterized by higher mRNA levels in these cases, except for CALR expression in blood, where a higher value was observed in the normal group, but these differences were not statistically significant (*p* > 0.05).

In the deformed group, the relative expression of Akt1s1 in liver and brain was statistically significantly higher than in the normal group (*p* < 0.05). COL1A2 exhibited the highest significant expression in blood of normal fish compared to the deformed group (*p* < 0.05). At the same time, the deformed group showed significantly higher mRNA levels in the heart. Despite differences in relative expression levels of the CHD, GH and SIX3 genes, no statistically significant differences were found (*p* > 0.05). In the case of IGF1 expression, there was a statistically significant difference (*p* < 0.05) in the heart, where the normal group displayed highly elevated expression levels compared to the deformed group. MSTN mRNA levels in the blood revealed higher values in the deformed group with statistically significant differences (*p* < 0.05). However, the mRNA levels of the deformed group were statistically significantly lower in the brain.

In the case of the CRH gene involved in stress response and the TLR2 gene linked to the immune system, diverse mRNA levels were also observed in different tissues (Fig 1). The mRNA levels of CRH in the normal group were lower than or equal to that in the deformed group in most cases, except for blood and brain tissues, where they were statistically significantly higher (*p* < 0.05). The TLR2 gene was overexpressed in the deformed group in all tissues, except abdominal fat, where it was equal to the mRNA levels of both groups. However, we measured a statistically significant difference (*p* < 0.05) for this gene only in the brain.

### Relationship between the level of relative gene expression and spinal column element content

Based on our findings, no significant difference was observed between the deformed and normal groups in regards to their macro- and microelement contents (*p* < 0.05). Table 3 shows the significant results of the linear regression and Spearman’s correlation between mRNA levels and macroelement contents. There was a detectable association between the expression of each gene and macroelement content in blood, heart, liver, dorsal muscle, and brain tissues. In each case, there was a strong correlation between the variables (Table 3). The explanatory power of the linear regression model was also strong in most cases.

**Table 3:** The result of significant correlations between mRNA levels and spinal macroelement contents based on linear regression and Spearman’s correlation test.

				Linear Regression					Spearman	
Tissue	Gene	Element	Group	p-value	R	R Square	Adjusted R Square	β	rho Correlation Coefficient	p-value
blood	CRH	Na	deformed						-0.886	0.019
blood	CALR	S	normal						0.886	0.019
blood	COL1A2	S	normal						0.886	0.019
blood	TLR2	S	normal						0.886	0.019
heart	Akt1s1	Mg	normal	0.018	0.890	0.792	0.740			
heart	IGF1	Mg	deformed						0.812	0.050
heart	IGF1	Mg	normal						-0.829	0.042
heart	CRH	Mg	normal						-0.943	0.005
heart	GH	Mg	normal						0.943	0.005
heart	MSTN	Mg	normal						-1.000	0.000
heart	TLR2	Mg	normal						-1.000	0.000
heart	CHD	Mg	normal						-1.000	0.000
heart	COL1A2	Mg	normal						-1.000	0.000
heart	CALR	Mg	normal						-1.000	0.000
heart	SIX3	Mg	normal						-1.000	0.000
heart	IGF1	P	deformed						0.812	0.050
heart	IGF1	S	deformed						-0.899	0.015
liver	TLR2	Mg	normal	0.002	0.963	0.927	0.909			
liver	SIX3	Mg	normal	0.009	0.919	0.845	0.807			
liver	COL1A2	Mg	normal	0.006	0.936	0.875	0.844			
liver	CHD	Mg	normal	0.015	0.900	0.809	0.762			
liver	CALR	Mg	normal	0.013	0.904	0.817	0.771			
liver	MSTN	Mg	normal	0.005	0.942	0.888	0.860			
liver	Akt1s1	Mg	normal						0.943	0.005
liver	IGF1	Ca	deformed	0.048	0.815	0.664	0.580			
dorsal muscle	GH	Mg	deformed	0.037	0.838	0.703	0.629			
dorsal muscle	CRH	Mg	deformed	0.049	0.814	0.663	0.578			
dorsal muscle	MSTN	K	normal						0.829	0.042
dorsal muscle	COL1A2	K	normal						0.886	0.019
brain	MSTN	Na	deformed	0.016	0.894	0.799	0.749			
brain	Akt1s1	Na	deformed	0.040	0.831	0.691	0.613			
brain	CALR	Na	deformed	0.039	0.834	0.696	0.620			
brain	CHD	Na	deformed	0.020	0.881	0.776	0.720			
brain	COL1A2	Na	deformed	0.039	0.835	0.697	0.621			
brain	CRH	Na	deformed	0.032	0.849	0.721	0.651			
brain	GH	Na	deformed	0.037	0.839	0.704	0.631			

Gray shading indicates those cases where there was significant difference between mRNA levels of the normal and deformed groups. p ≤ 0.05.

In the blood, CRH expression showed a strong negative correlation (r = -0.886, p = 0.019) with Na in the deformed group. Also in the blood, there were a strong positive correlation between the S contents and mRNA levels of the CALR (r = 0.886, p = 0.019), COL1A2 (r = 0.886, p = 0.019) and TLR2 (r = 0.886, p = 0.019) genes in the normal group. In the heart of deformed fish, the Mg content showed a relatively strong positive correlation (r = 0.812, p = 0.05) with the IGF1 mRNA level, whereas the same relationship was characterized by a strong negative correlation (r = -0.829, p = 0.042) in the normal group. The Mg contents were characterized by a strong negative correlation for most genes (CRH: r = -0.943, p = 0.005; MSTN, TLR2, CHD, COL1A2, CALR and SIX3: = -1,000, p = 0.000) in the heart tissue of the normal group. Exceptions were the GH (r = 0.943; p = 0.005) and Akt1s1 (R = 0.890, p = 0.018) genes, where strong positive correlation and regression were measured. Furthermore, the P (r = 0.812; p = 0.05) and S (r = -0.899; p = 0.015) contents also showed a significant correlation with IGF1 mRNA levels in the heart tissue. The Mg contents of liver showed a strong positive correlation with mRNA levels of TLR2 (r = 0.963, p = 0.002), SIX3 (r = 0.919, p = 0.009), COL1A2 (r = 0.936, p = 0.006), CHD (r = 0.900, p = 0.015), CALR (r = 0.904, p = 0.013), MSTN (r = 0.942, p = 0.005) and Akt1s1 (r = 0.943, p = 0.005) genes in the normal group. In the liver tissue of the deformed group, there was a detectable positive linear relationship in the relation of IGF1/Ca (r = 0.815, p = 0.048). In the dorsal muscle, Mg contents in the deformed group showed a significant positive linear relationship with the mRNA levels of the GH (r = 0.838, p = 0.037) and CRH (R = 0.814, p = 0.049) genes, whereas K showed a strong positive correlation with expression levels of MSTN (r = 0.829, p = 0.042) and COL1A2 (r = 0.886, p = 0.019) genes in the normal group. Finally, only Na content showed a significant linear relationship in the brain for several genes (MSTN: r = 0.894, p = 0.016; Akt1s1: r = 0.831, p = 0.040; CALR: r = 0.834, p = 0.039; CHD: r = 0.881, p = 0.020; COL1A2: r = 0.835, p = 0.039; CRH: r = 0.849, p = 0.032; GH: r = 0.839, p = 0.037), and this was characteristic of the deformed group in all cases.

Table 4 presents the significant results of the relationship between mRNA levels and microelement contents. In the blood, only Fe content was found to be associated with CRH gene expression, which was a significant negative correlation (r = -0.886; p = 0.019). In the liver, the IGF1 mRNA level of the deformed fish showed a strong positive relationship with the following elements: Ba (r = 0.857, p = 0.029), Mn (r = 0.944, p = 0.005), Sr (r = 0.841, p = 0.036), Cu (r = 0.943, p = 0.005). Similarly, a significant positive correlation was estimated between TLR2 and Cu (r = 0.829, p = 0.042), and CRH also showed a similar positive correlation with Ba content, but in the normal fish group (r = 0.818, p = 0.047). In the genitals, Fe content in normal fish was associated with the expression of the CHD (r = 0.827, p = 0.042) and CRH (r = 0.828, p = 0.042) genes. In the same tissue, Mn content showed a strong negative correlation with the MTSN and CALR genes (r = -0.829; p = 0.042 and r = -0.943; p = 0.005) in the deformed group. In the dorsal muscle, Mn content was also associated with the expression of COL1A2 (r = 0.872, p = 0.023) and TLR2 (r = 0.860, p = 0.028) genes. Finally, brain tissue was affected in the most cases of relationships between gene expression and microelement content. In all cases, Fe content was the associated microelement and this association was detected in the deformed group (MSTN: r = 0.951, p = 0.004; Akt1s1: r = 0.893, p = 0.016, CALR: r = 0.873, p = 0.023, CHD: r = 0.932, p = 0.007, COL1A2: r = 0.883, p = 0.020, CRH: r = 0.926, p = 0.008, GH: r = 0.886, p = 0.019; SIX3: r = 0.824, p = 0.044).

**Table 4:** The result of significant correlations between mRNA levels and spinal microelement contents based on linear regression and Spearman’s correlation test.

				Linear Regression					Spearman		
Tissue	Gene	Element	Group	p-value	R	R Square	Adjusted R Square	β	rho Correlation Coefficient	p-value	
blood	CRH	Fe	deformed						-0,886	0,019	
liver	CRH	Ba	normal	0.047	0.818	0.669	0.586				
liver	IGF1	Ba	deformed	0.029	0.857	0.735	0.668				
liver	IGF1	Mn	deformed	0.005	0.944	0.891	0.863				
liver	IGF1	Sr	deformed	0.036	0.841	0.707	0.633				
liver	IGF1	Cu	deformed						0.943	0.005	
liver	TLR2	Cu	deformed						0.829	0.042	
genitals	CHD	Fe	normal	0.042	0.827	0.684	0.605				
genitals	CRH	Fe	normal	0.042	0.828	0.686	0.607				
genitals	MSTN	Mn	deformed						-0.829	0.042	
genitals	CALR	Mn	deformed						-0.943	0.005	
dorsal muscle	COL1A2	Mn	deformed	0.023	0.872	0.761	0.701				
dorsal muscle	TLR2	Mn	normal	0.028	0.860	0.740	0.674				
brain	MSTN	Fe	deformed	0.004	0.951	0.904	0.880				
brain	Akt1s1	Fe	deformed	0.016	0.893	0.798	0.747				
brain	CALR	Fe	deformed	0.023	0.873	0.762	0.703				
brain	CHD	Fe	deformed	0.007	0.932	0.868	0.835				
brain	COL1A2	Fe	deformed	0.020	0.883	0.780	0.725				
brain	CRH	Fe	deformed	0.008	0.926	0.858	0.822				
brain	GH	Fe	deformed	0.019	0.886	0.785	0.731				
brain	SIX3	Fe	deformed	0.044	0.824	0.679	0.599				

Gray shading indicates those cases where there was significant difference between mRNA levels of normal and deformed groups. p≤0.05.

Based on the results, the expression of the CRH gene in deformed fish is associated with lower Na and Fe contents in the blood. With higher expression of COL1A2 gene in the blood of normal fish, the S content also increases. The COL1A2 gene showed lower relative expression levels in heart tissue and thus had a significant negative correlation with Mg content. A strong correlation between IGF1 mRNA level and Mg content in the heart can be hypothesized, which was confirmed by the Spearman correlation in both study groups. On this basis, deformed fish are characterized by lower IGF1 expression associated with higher Mg contents. In parallel with the lower IGF1 mRNA level of the deformed group, a positive correlation with P content and a negative one with S content were estimated. The relative expression of Akt1s1 gene was significantly lower in the liver, and in this context, Mg content showed a strong positive correlation in the normal group. Lower mRNA levels of MSTN and CRH genes in the brain of the deformed group showed a significant linear relationship with Na and Fe contents, whereas the same was associated with higher relative expression levels of the deformed group in the case of the Akt1s1 gene.

## Discussion

The present study describes the relative expression levels of different growth- and stress-related genes in connection with the spinal deformity of common carp in normal and deformed groups, in different types of tissues. Furthermore, correlations between mRNA levels and the macro- and microelement contents of the spinal cord were also examined. We found statistically significant connection in 11 out of 15 cases in the deformed group, in which the Akt1s1, COL1A2, CRH, IGF1 and MSTN genes were involved. Grouped by tissue, in six cases the brain, in five cases the heart, in three cases the blood, and in one further case the liver was involved. Finally, the macroelements playing a part in these associations were Na: 4 times, Mg: 4 times, S: twice, P: once, and the microelement Fe: 4 times. In the following, we discussed these relationships in detail.

### Akt1s1

The function of skeletal muscle cells, also known as myocytes, affects the function of Akt1s1, resulting in increased phosphorylation, leading to increased levels of Akt1s1 regulators [83]. Higher relative expression levels of the Akt1s1 gene in brain tissue samples from the deformed group showed a strong positive regression with the Na and Fe content of the spine. The Na content plays an important role in the transmission of nerve impulses and in membrane transport processes, therefore it strongly affects brain function. In its absence, muscle atrophy can occur. The transporter function is strictly dependent on Na^+^ [84].

The Akt1s1 gene and the elements Na and Fe also affect the nervous system, and a relationship between their amounts measured in the brain and the spine has been revealed in individuals with deformities. This suggests a key role for the nervous system in the development of spinal deformity, and raises the possibility of further studies to elucidate the direction and exact mechanism of the relationship.

In general, the Mg content of the spine was strongly negatively correlated with the mRNA levels of most genes (CALR, CHD-like, COL1A2, CRH, MSTN, TLR2 and SIX3) in the heart tissues of the normal group. Exceptions were the GH and Akt1s1 genes, where strong positive correlation and regression were measured. Because Mg is primarily present in bones and muscles, it is understood that its concentration is more related to the GH and Akt1s1 genes. The relative expression of Akt1s1 in the liver of the normal group was significantly lower. Furthermore, the Mg content of the spine showed a strong positive correlation with the Akt1s1 mRNA level in the normal group. The liver is a key component of the excretory system. Our results suggest that the expression of Akt1s1 in the liver may have an effect on the accumulation of Mg in the spine and that this regulatory role may also be important in the development of the spinal deformity of the common carp.

### COL1A2

Based on our results, COL1A2 was expressed at significantly higher levels in the blood of normal fish than in the deformed group. Degradation of the ECM (extracellular matrix) leads to muscle damage caused by structural and functional changes to myocytes [85]. Downregulated COL1A2 caused atrophy in zebrafish (*Danio rerio*), because among other things, it participates in the regulation of the organization of the extracellular matrix and the development of the skeletal system [86]. The loss of collagen and fibroblast function, and the increase in matrix metalloproteinase activity can promote the degradation of the ECM, leading to cell shrinkage and senescence [86]. Thus, the lower mRNA level of the COL1A2 gene in the blood of deformed fish is another indication that this gene may play a key role in the development of pathological structural changes in fish. The sulfur content of the spine was strongly positively correlated with a significantly higher mRNA level of COL1A2 in the blood of the normal group. Both collagen and sulfur are important components of tissue strength, and our results suggest that adequate expression of collagen in the blood, as an important element in sulfur transport, may have a direct effect on tissue sulfur content and thus on normal cell development.

In our study, expression of COL1A2 gene in the heart tissue of the deformed group was significantly upregulated compared with the normal group. Moreover, there was a very strong negative correlation in the heart of normal fish between the COL1A2 mRNA level and the Mg content of spine. Since both the heart and the spinal bone tissue are also involved in the body’s Mg turnover, it would be worthwhile to further investigate and clarify its direction and exact significance in the development of spinal deformity.

### CRH

In our study we were able to show a significant difference in the CRH expression in the brain and blood of the two groups. In both tissues, the gene had higher activity in the normal group. An interesting pattern was also observed in further analyzes. In both tissues, CRH mRNA levels were associated with spinal Na and Fe content and this was observed in all cases in the deformed group. The levels of both elements measured in the spine were strongly negatively correlated with the mRNA level of the blood. In the brain, these element contents also showed strong regression with CRH expression. As explained above, one of the most important physiological roles of Na is in the transmission of nerve impulses and in membrane transport processes, and therefore Na content strongly influences brain function. Furthermore, iron also has a major effect on the nervous system in the electron transport, the synthesis and packaging of neurotransmitters, their uptake and their degradation into other iron-containing proteins, through which they can directly or indirectly alter brain function [71]. Given these facts, our results are in line with expectations; however, we also demonstrate the involvement of these regulatory factors (CRH gene, Na and Fe elements) in spinal deformation of carps.

### IGF1

In the present study, we measured statistically significant differences between the mRNA levels of IGF1 of the two groups, but only in the heart, where the deformed group was characterized by lower gene expression. Skeletal muscle mass depends on the balance between the rates of protein synthesis and degradation. IGF plays a key role in protein synthesis [87]. Protein synthesis depends on intracellular mTOR signaling, which is activated by insulin/IGF1-PI3K-Akt cascade phosphorylation signals [88, 89]. The genetic inactivation of IGF1R or mTOR in the muscle can result in decreased muscle fiber size or muscular dysplasia [90, 91]. The key to repairing defects of the peripheral nervous system is to promote the regeneration of axons and prevent the atrophy of muscles under the control of the nervous system [92]. During such repair, axon guidance molecules play an important role in the orderly growth of axons [93]. Chen-Chen et al. [87] found downregulation of key factors–which also included IGF1 –relating to axon guidance, which indicates that axons may be unable to form in an orderly manner during or after excessive exercise, further contributing to muscle atrophy. Thus, the significantly lower IGF1 expression we measured in the hearts of deformed individuals may indicate heart muscle atrophy. At the same time, it is an indication of the extent of spinal deformity which affects the entire body of an animal, and what adverse effects it can have on key systems.

The phosphorus content of the spine was strongly positively correlated with the lower IGF1 mRNA level of the heart in the deformed group, whereas the sulfur content of the spine was strongly negatively correlated in the same group and the same tissue. Based on the above, it is not unexpected that we found a relationship between factors involved in growth and energy turnover and spinal deformation. What was more unexpected, however, was that all of this was identified in the heart. This again justifies the complex background of spinal deformity and its effect on the whole organism.

A remarkable result of our study was a strong positive correlation between heart IGF1 mRNA level and spinal Mg content in the deformed group. However, a strong negative correlation characterized the same relationship in the normal group. In the present study, this is the only case where we obtain a statistically significant result in the same relationship in both groups. Structural damage to muscle cells could be linked to magnesium. Correlations between IGF1 concentration and magnesium and lipids could be used as biomarkers for early diagnosis of cardiovascular disease. Patients with diabetes have lower magnesium levels compared to control patients, as IGF1 is able to increase intracellular magnesium levels and also to reverse the dull response of hypertensive cells to insulin [94]. IGF1 signaling regulates cardiac contractility, metabolism, hypertrophy, autophagy, aging, and apoptosis [95]. These symptoms may also appear in connection with spinal deformity, thus there can be no doubt about the serious role of Mg in the development of the pathological lesion. The heart muscle is indirectly connected to all parts of the body through circulation. Thus, the expression of IGF1 as a key regulatory gene in cell growth in this tissue may also have an indirect effect on tissues directly involved in deformation. In our opinion, the relationship demonstrated in both groups is a mutually reinforcing result, and the factors examined in this context may also be key factors in the phenomenon of spinal deformity due to genetic reasons. Thus, the heart tissue, the IGF1 gene, and the Mg content of the spine may be a system worthy of further, more detailed study.

### MSTN

The MSTN gene was expressed at a significantly lower level in the brain of the deformed group. The role of MSTN in the brain has not been elucidated for a long time. However, in addition to influencing skeletal muscle mass, it also plays a very important role in brain function [96, 97]. MSTN inhibits myoblast proliferation and differentiation, it regulates myogenesis. Expression of myostatin in brain regions may indicate that it acts as a specific regulator of neurogenesis in the region [98]. Thus, it cannot be ruled out by the regulatory function that the underactive gene in the brain may be directly related to the development of spinal deformity in carp. A common complication of cerebral ischemia is loss of skeleton and muscle mass, which is associated with myostatin. This was supported by an increase in MSTN expression in the skeletal muscle of the paretic limb compared to the non-paretic limb in stroke patients [99]. These results also support the assumption that myostatin expression may be associated with brain function, which may affect skeletal muscle abnormal development.

The complete MSTN gene knockout fish showed a shorter body length, the skull structure was smaller, and osteosarcomas were found in the individuals in the experiment reported by [100]. The spinal nerves of genome-engineered individuals were much more perpendicular than those of the wild type, therefore these fish became taller. Although the vertebrae were also shorter and smaller, no lordosis or scoliosis was observed [100]. Although we did not perform morphometric measurements on the fish studied in the present work, we believe that our results represent another addition and support the prominent importance of MSTN in the spinal deformity of fish.

We found a statistically significant, strong regression between the lower mRNA level of the brain of the deformed group and the Na and Fe content of the spine. Sodium is an essential element for the integrity and functionality of the animal body, because it is the principal cation in extracellular fluids [62]. Fe is required for the proper myelination of the spinal cord and the white matter of cerebellar folds in the brain, and is a cofactor for a number of enzymes involved in neurotransmitter synthesis and processing which may directly or indirectly alter brain function [71, 101]. Statistically significant regression in the deformed spine between Na and MSTN, furthermore Fe and MSTN–all of which play a role in nerve regulation–suggests that this detrimental phenomenon can be traced back to causes rooted in the nervous system. At the same time, it highlights that the background of the phenomenon is complex and multi-factorial, but its precise understanding requires further investigation.

Gene knockout experiments may support studies on gene expression. In aquaculture, it is mainly used in connection with the development of skeletal muscle, therefore, validating our results with such methods can be a logical continuation of our study. Such results are not available for many genes, but these are available for example in the case of the myostatin gene, as fast-growing individuals have become important for production. For example, Yeh et al. [102] used the CRISPR/Cas9 method to mutate the myostatin gene in Japanese medaka fish (*Oryzias latipes*). Outcome and heritability have been studied in several generations. The body length and weight of the fish also increased significantly, and muscle hypertrophy occurred. Spinal deformities have also developed in some individuals. Because MSTN activates the Smad2/3 pathway, which in turn leads to down-regulation of MRFs (myogenic factors), the expression of Smad2/3-regulated MRFs [102] has been studied in one generation. Three major myogene-related factors were subjected to real-time PCR to detect their expression: Myf5, Myogenin, and MyoD22, 27. From the post-juvenile stage, expression levels in the factors were significantly elevated. These results do not show any direct association between MSTN rash and the Smad pathway, however, they concluded that within 4 weeks of hatching, the function of the Smad 2/3 pathway is relatively suppressed [102]. During the experiment, some of the individuals in the F4 generation showed severe spinal deformities and were therefore tested. All deformed individuals tested showed a homozygous 22 bp insertion at the target site [102]. In fish farming, these studies are not too common yet for different commodity fish species. Kishimoto et al. demonstrated [100] the creation of a new species of fully knockout red sea bream (*Pagrus major*). The mutations were created by deletions of the first exon of MSTN, which cause disruption of the C-terminal active domain of MSTN. No high degree of spinal deformity was observed in the individuals during the experiment. In 2022, Shahi et al. [103] studied the improvement of skeletal muscle and growth in economically important carp *(Cyprinus carpio*) without the introduction of external DNA. In their study, sgRNA/Cas9 ribonucleoprotein (RNP) was used to knock out the carp MSTN gene (KO) and subsequently monitored for hatchability, survival, body deformities, and growth performance. Individuals were observed for 90 days after hatching, showed a significant increase in body weight and no deformity. Overall, expression levels of myogenic regulatory factors (MRF), myogenic factor 5 (myf5), and myogenin (myog) mRNA were upwardly regulated in carp. The study shows that CRISPR/Cas9-based RNPs can be effectively used to target the MSTN gene in common carp to increase skeletal muscle mass [103].

## Conclusions

In the present study, we identified a statistically significant relationship in the group of normal fish in 4 cases and in the group of deformed fish in 11 cases. This suggests serious physiological differences between healthy and deformed fish.

The CRH (4) and IGF1 (4) genes were involved in most of the identified contexts. The former plays a key role in hormonal, autonomic responses to physiological and behavioral stress in vertebrates [104], whereas the latter is an important component of the hypothalamic-pituitary-somatotropic axis [46], and as such, it plays a key role in regulating growth.

Of the tissues examined, brain (6), heart (5), and blood (3) occurred most frequently in a significant relationship in relation to gene expression and spinal element content.

In the vast majority of cases, the Na (4) and Mg (4) macroelement content of the spine as well as its Fe (4) microelement content were correlated with mRNA levels measured in different tissues. These regression and correlation relationships were always strong and statistically significant. The most important biological functions of these elements can also be related to the functioning of the nervous system.

No statistically significant difference was detected between the two study groups in the tissue directly affected by the deformity, in the dorsal muscle, or in the GH gene, which is the fundamental determinant of growth. In any case, this reinforces the hypothesis that the development of spinal deformity may not be based solely on local effects, and its consequences have an impact on the whole organism. For this reason, it is correct to address spinal deformity not merely as an aesthetic defect, but as a negative phenomenon that must be prevented or reduced for both animal welfare and economic reasons.

In conclusion, mRNA levels of the genes and elemental levels involved in the regulation of the nervous system and growth were most closely related in individuals with deformed spine. However, our study was only intended to reveal the relationships. Exploring the direction and exact background of the relationships will be the task of further studies. In any case, it seems certain that the regulatory role of several genes and elements is simultaneously involved in the background of the phenomenon. We believe that the present results support our notion that there are complex phenomena behind spinal deformity and their precise exploration requires a holistic approach.

## Supporting information

S1 Table Macro-and microelement content of spine of studied common carps. (XLSX)

S2 Table Average Ct values of studied genes per tissue. (XLSX)
